# Status of human dignity of adult patients admitted to hospitals of Tehran 

**Published:** 2014-12-01

**Authors:** Fariba Borhani, Abbas Abbaszadeh, Soolmaz Moosavi

**Affiliations:** 1 Assistant Professor, Department of Nursing Ethics, Medical Ethics and Law Research Center, Shahid Beheshti University of Medical Sciences, Tehran, Iran;; 2 Professor, School of Nursing and Midwifery, Shahid Beheshti University of Medical Sciences, Tehran, Iran;; 3 MSc, Medical Ethics and Law Research Center, Shahid Beheshti University of Medical Sciences, Tehran, Iran.

**Keywords:** patient dignity, nursing care, nurse-patient relationship

## Abstract

Maintaining dignity and respect is among patients’ most fundamental rights. The importance of patient dignity, the status quo, patients’ needs, and a shortage of survey studies in this area were the underlying incentives for conducting this study.

This was a cross-sectional descriptive study in which data were collected through Patient Dignity Inventory (PDI). The questionnaire was completed by 280 inpatients in 2012 to determine their perspectives on their personal state of human dignity.

In this study, the mean score of patients’ dignity was 1.89 out of 5 (SD = 0.81). Results indicated a significant relationship between type of hospital and the distress caused by disease symptoms, peace of mind, and social support (*P* < 0.05). There were also relationship between type of ward and dependency (*P* < 0.05), type of disease and dependency (*P* < 0.05), gender and social support (*P* < 0.05), household size and peace of mind (*P* < 0.05). The person’s satisfaction with household income showed significant relationship with symptom distress, dependency and existential distress (*P* < 0.05). Results showed a significant inverse correlation between age and patient dignity (*P* = 0.005, r = - 0.166). However, the relationship between employment status, health insurance, education level and the above factors were insignificant. Studies indicate that there is a relationship between patients’ dignity and mental distress, and therefore policy makers and health services officials should establish and implement plans to maintain and enhance patients’ dignity in hospitals. Educating the health team, particularly the nurses can be very effective in maintaining patients’ dignity and respect.

## Introduction

Dignity is a complex, yet important issue in patient care that has many dimensions rooted in emotions, behavior, appearance, and privacy (1). For many patients, dignity and respect is the last and most significant thing that they may request of their relatives and caregivers, especially if their disease is acute and life-threatening (2). Today, important issues associated with spiritual health and respect for patients’ human status and dignity are becoming the main concept in care centers, particularly in adult care departments, nursing homes and hospices (3).

Human dignity and respect is a complex, vague and multi-faceted concept interrelated with respect for patients’ wishes, maintaining their privacy, self-esteem and control, reducing their shame, and similar issues (4, 5). Although these concepts are an important part of our daily lives, people are not comfortable talking about them (4). Simply put, dignity is an inherent and non-negating value that a person possesses by virtue of being human. This value is manifested in a person’s existence and in relationship with others. Illness, disability, neediness, reduced power and choice, decreased privacy, treatment, palliation and hospitalization can all affect a person’s dignity. Limiting patients’ dignity can affect their body, spirit, mood, and spirituality, and expose them to stress (6).

In literature there is reference to five behavioral standards in health center staff that are indicators of their regard for patients and a high quality of care. These five standards include: a) respect, meaning that all hospital staff should respect patients in all circumstances; b) attitude, meaning that all hospital personnel should exhibit a positive attitude toward their patients; c) behavior, meaning that the entire treatment team should exhibit professional behavior in dealing with patients; d) communication, meaning that the entire treatment team should communicate with patients in a manner that expresses sensitivity to their needs and preferences; and e) privacy and dignity, meaning that the entire staff must maintain patients’ privacy and dignity in all circumstances (7).

Nursing literature frequently observes that dignity is highly valued by patients (7). Taking a humanistic approach, nursing theorists consider respect for patients and maintaining their dignity as central and of high priority in nursing care. They also assert that maintaining patient dignity is among nurses’ fundamental roles and may even be more important than providing health care (4). Nevertheless, there are many issues related to the subject of patient dignity that remain unresolved and require clarification (7).

Studies conducted on patient dignity define it as a feeling of peace, control and value, and a special type of behavior. Circumstances that can negatively affect patient dignity include loss of privacy, and the hospital staff’s commanding and domineering behavior. Furthermore, creating an atmosphere that enhances patient dignity can improve a culture of respect for patients (8).

Many qualitative and quantitative studies have examined the subject of patient dignity. In qualitative studies that attempted to explain participants’ perspectives on patient dignity among the nursing home elderly, the themes included being ignored, fragility and dependence, inner strength, and togetherness (9). In another qualitative study conducted by forming an elderly focus group, attributes such as kindness and sympathy, respect for human values, and observing patients’ rights were found to be important in preserving dignity (2).

Likewise, results of Dwyer et al study using qualitative content analysis revealed that to enhance the dignity of the elderly, nurses require commitment, supervision and training. Based on the findings of the same study, nurses are preoccupied with what they can do to maintain patient dignity and perform what is expected of them (9).

To assess the level of human dignity experienced by patients hospitalized in various wards, Chochinov et al. created a tool that has also been used by many subsequent studies in this area. In the model adopted by these studies, the patient dignity construct is assessed in three categories: dignity in relation to the disease, actions for maintaining dignity and respect, and social respect. These studies have generally reported threatened patient dignity (10). A cross-sectional study found that 7.5% of patients in end-of-life stages experience an intense lack of dignity. A follow-up of these patients over a 6-month period revealed that compared to other patients, this group exhibited more mental distress, physical stress, dependency, and disinterest in life. These findings demonstrate the close relationship between human dignity and a variety of distresses in patients (11).

A review of the studies conducted on the subject in Iran found that they were largely theoretical and review studies, and none had used any particular tools to assess patient dignity. In a study titled “Dignity in Medicine”, Avizhgan and Mirshahjafari stressed the importance of maintaining patient dignity in the last stages of life through patient-oriented communication. The above-mentioned study asserted that this communication must involve telling the truth, giving the patient correct information, an appropriate method of conveying bad news, maintaining privacy, confidentiality, reliability, the right to choose and decide, dealing with inappropriate treatment demands, euthanasia, and unconditional respect (12). Similarly, Sadeghi and Dehghan Nayeri stated that observing patient dignity and respect is a patient’s right and comprises maintaining privacy, confidentiality, and non-exposure (13).

Considering the importance of patient dignity and a shortage of survey studies in this area that indicate the current state of affairs and patient needs, the present study was conducted to examine the issue further.

## Method

This was a cross-sectional descriptive study conducted in Tehran during 2013 to investigate the perspectives of adult patients in hospitals affiliated with Shahid Beheshti University of Medical Sciences on their personal state of human dignity. Sample size was calculated based on a pilot study, and considering mean, standard deviation (0.42), and 0.05 error, 280 patients were selected from surgical and internal medicine wards. Quotas were based on the number of hospital beds, and patient availability power associated with the determined sample size was 0.80. Study exclusion criteria were: inability to speak Persian, absence of full consciousness, mental problems, and lack of physical readiness.

Data were collected using Patient Dignity Inventory (PDI) designed by Chochinov et al. (10) in 2008 to measure the sources of distress associated with patient dignity. The questionnaire was translated into Persian using the backward-forward method. Its content validity was confirmed by 10 nursing faculty members, and its reliability was determined in a pilot study of 19 patients through internal consistency of 0.87 (Cronbach's alpha). This questionnaire contained 25 items including five factors: symptom distress (items 3, 5 - 9), peace of mind (items 15 - 17), dependency (items 1, 2, 20), social support (items 21, 22 - 25), and existential distress (items 4, 11 - 14, 18). Given the number of items in the questionnaire (25 items), and the 5-point Likert scale responses, total score ranged from 25 to 125, and mean score ranged between 1 and 5. Scoring in previous studies (10, 14) had been as follows: not a problem (1), a slight problem (2), a problem (3), a major problem (4), an overwhelming problem (5). The items of PDI have negative load (written negatively), so higher scores mean more problems associated with dignity.

Samples were selected using convenient sampling method and all eligible patients completed the questionnaire. The questionnaire was completed as self-report by patients, and illiterate patients were assisted by one of the researchers. The study proposal was approved by the Ethics Committee of the Research and Technology Department at Shahid Beheshti University of Medical Sciences in March 2013. Participants were informed of the study objectives, voluntary participation, data confidentiality and anonymity, and were assured that non-participation would in no way interfere with their treatment and care. 

Data analysis was performed using SPSS 18 statistical software. Frequency, percentage frequency, mean and standard deviation were used to identify descriptive parameters, and t-test, ANOVA and Pearson’s correlation coefficient were used for the analytical parameters.

## Results

Data were collected from four general hospitals affiliated with Shahid Beheshti University of Medical Sciences in the department of internal medicine (neurology, nephrology, endocrinology, gastroenterology, pulmonary, obstetrics, cardiology, etc.) and the department of surgery (orthopedics, neurosurgery, vascular surgery, urology, etc.).

The majority of the study populations were men (59.6%), and women comprised 40.4% of the participants. Patients’ mean age was 46.9 (SD = 16.7), ranging from 15 to 90 years. 

Table 1 presents the patients’ demographic details.

**Table 1:** Patients’ demographic details

**Variables**	**Mean (percent )**
* Marital Status	
Married	73.9 %
Single	17.9 %
Divorced	1.8 %
Widowed	6.4 %
* Employment Status	
Unemployed	10.7 %
Employed	45.4 %
Retired	12.5 %
Housewife	31.4 %
* Education Level	
Illiterate	22.5 %
High school diploma or higher	67.5 %
Bachelor's degree or higher	9.6%
* Disease Type	
Internal ward	49.3 %
Surgical ward	50.7 %
* Health Insurance	
Uninsured	26.8%
Covered	73.2%
* Satisfaction with Household Income	
Low	62.5 %
Average	35.8%
Increased	1.8%

The mean overall score of human dignity was 1.89 out of a possible 5 (a lower score indicating a better evaluation), and 2.09 (SD = 0.92) for symptom distress, 1.88 (SD = 0.96) for existential distress, 1.89 (SD = 1.01) for dependency, 1.91 (SD = 0.97) for peace of mind, and 1.60 (SD = 0.89) for social support dimensions. Table 2 presents the relationships between demographic details and the dimensions of dignity.

**Table 2:** The relationship between the dimensions of human dignity and demographic characteristics

**Variables**	**Social Support**	**Peace of Mind**	**Existential Distress**	**Symptom Distress**	**Dependency**
Hospitals	*P=0.004	*P=0.003	*P=0.008	*P* =0.204	*P*=0.329
Type of Ward	*P*=0.984	*P*=0.984	*P*=0.710	*P*=0.786	*P=0.006
Gender	*P=0.031	*P*=0.203	*P*=0.767	*P*=0.083	*P*=0.966
Type of Disease	*P*=0.346	*P*=0.807	*P*=0.83	*P*=0.790	*P=0.049
Insurance Status	*P*=0.91	*P*=0.9	*P*=0.87	*P*=0.22	*P*=0.54
Household Size	*P*=0.21	*P=0.004	*P=0.02	*P*=0.96	*P*=0.31
Satisfaction with Household Income	*P*=0.05	*P*=0.05	*P=0.001	*P=0.000	*P=0.005
Education Level	*P*=0.442	*P*=0.833	*P*=0.816	*P*=0.083	*P*=0.434
Marital Status	*P*=0.801	*P*=0.510	*P*=0.891	*P*=0.828	*P*=0.196
Employment Status	*P*=0.215	*P*=0.555	*P*=0.345	*P*=0.078	*P*=0.466

Tables (2 and 3) reports a significant relationship between hospitals and existential distress, peace of mind, and social support. Significant relationships also existed between type of ward and dependency, type of disease and dependency, gender and social support, household size and peace of mind and existential distress. The relationships between satisfaction with household income and symptom distress, dependency and existential distress were significant.Mean of human dignity and its dimensions in patients participating in the study based on demographic variables are shown in table 3. The relationships between age and peace of mind, and symptom distress were significant (table 4). No significant relationship was found between employment status, insurance status or education level and the five factors (table 2).

**Table 3:** The mean of human dignity and its dimensions based on demographic variables

**Variables**	**Mean ± SD**	**Peace of Mind**	**Symptom Distress**	**Existential Distress**	**Social Support**	**Dependency**
Gender Female Male	1/90 ± 0/61 1/88 ± 0.87	t = 1.62 df = 1 *P*= 0.20	t = 3.06 *P* = 0.08	t = 0.08 *P* = 0.76	t = 4.68 **P = 0.03	t = 0 *P* = 0.99
Ward type Internal Surgical	1/68 ± 0/72 1/91 ± 0/86	t = 0 df = 1 *P* = 0.98	t = 0.07 *P* = 0.78	t = 0.13 *P* = 0.71	t = 0.22 *P* = 0.63	t = 7.8 **P = 0.006
Hospital A B C D	2/1 ± 0/91 2/09 ± 0/91 1/85 ± 0/83 2/26 ± 0/82	f = 4.76 df = 3 **P = 0.003	f = 1.54 *P* = 0.20	f = 4.01 **P = 0.008	f = 4.52 **P = 0.004	f = 1.15 *P* = 0.32
Household Size < 5 people > 5 people	1/86 ± 0/77 2/09 ± 1/08	t = 8.49 df =1 **P = 0.004	t = 0.002 *P* = 0.96	t = 5.11 *P* = 0.025	t = 1.54 *P* = 0.21	t = 1.01 *P* = 0.31
Satisfaction with Household Income Low Average Increased	2/04 ± 0/85 1/65 ± 0/66 1/41 ± 0/33	f = 2.91 df = 2 *P* = 0.056	f = 8.12 **P = 0.000	f = 7.59 **P = 0.001	f = 2.99 *P* = 0.052	f = 5.46 **P = 0.005

* *P* < .05;

** *P* < 0.01;

*** *P *< 0.001

**Table 4:** Relationship between age and dimensions of human dignity

**Age**	**Social ** **Support**	**Peace of ** **Mind**	**Dependency**	**Existential ** **Distress**	**Symptom ** **Distress**	**Total** **Dignity**
**Dignity Dimension**						
Pearson’s Correlation Coefficient	- 0.099	- 0.215	- 0.115	- 0.115	- 0.166	- 0.169
*P*-value	0.09	*0.000	0.05	0.05	*0.005	*0.005

According to the table 4, patient dignity was lower in older people. 

## Discussion

The present study investigated patients’ perceptions of their personal state of human dignity. The results demonstrated that patient dignity was properly observed in the study hospitals with an overall mean score of 1.89 out of a possible 5 and a lower score indicating a better state of human dignity. A study by Hack et al. on cancer patients in Canada found that the majority of patients reported a suitable state of human dignity (15). However, other studies found an inappropriate state of human dignity (1, 4, 16). Participants in another study by Thornock and Kelleher conducted on ICU patients also reported a low sense of dignity, which may have resulted from these particular patients’ special conditions, including loss of control, autonomy and privacy, and lack of information and awareness. The decreased dignity of patients suffering from urological conditions in Baillie’s study could have resulted from loss of privacy, use of treatment equipment and catheters, and bodily exposure. Factors affecting human dignity may pertain to the hospital (environment and staff behavior) or the attributes and attitudes of patients (acceptance of the disease, rational thinking, mood or sense of humor). In this study, the good-feeling factor induced by the participating patients’ state of human dignity could be attributed to the fact that they were relatively young (46.9%), had been admitted to surgical and internal medicine wards, had health insurance (72.5%), and the majority did not suffer from critical conditions. 

Results also demonstrated that hospitals directly affect patient dignity. Patients in Shahid Modaress Hospital experienced a better state of dignity. The difference may be attributed to the hospitals’ physical and mental atmosphere. According to investigations conducted by the researcher, hygiene and sanitation, air conditioning, physical appearance and size of rooms were more favorable in Shahid Modaress Hospital compared to the other hospitals. 

Another factor that can affect patients’ perception of themselves is the manner in which they are perceived by other people. When people accompany a patient to the hospital, there is a change of atmosphere and everyone begins to adapt psychologically to the new conditions. Most patients experience less dignified feelings due to the fear of loss of control, autonomy, and personal space. Therefore, the physical and psychological environment of the hospital or a ward can reduce or increase feelings of dependence, peace of mind, and mental distress (4). Evidence demonstrates that factors such as poor location hygiene, noise, and lack of respect for privacy can threaten patient dignity. Conversely, treating patients respectfully and giving them information, respecting their right to choose, obtaining informed consent, involving patients in treatment and care decisions, an increased level of patient autonomy, and above all nurses’ attention to patients can enhance dignity (1, 17 - 20). Studies indicate that patients’ self-control and accountability significantly influence their daily activities and decisions, and that nurses play a particularly important role in maintaining these values in patients. Nurses can balance patients’ feelings of autonomy and dependence through understanding their needs and requirements, and by treating them respectfully (21).

Certain researchers assert that shortages in hospital facilities (personnel, space and equipment) may affect the dignity of the treatment team, making them feel disrespected within the organization and thus affecting their ability to maintain and enhance their patients’ dignity (4).

In the present study, type of disease correlated significantly with patient dignity (dependence factor), and patients in internal medicine wards were in a better state overall. This may be due to the fact that patients awaiting surgery face problems such as treatment costs, dependence on the treatment team and family members, loss of control, anxiety, and depression more than other patients. Previous studies have also found a relationship between type of disease and the status of dignity (4). Anxiety, fear, loss of sense of control, dependence, invasive procedures and anesthesia affect patients’ perceptions of themselves and their expectations of others, especially the treatment team (4). In the present study, a significant relationship was found between type of disease and dependence. The state of dignity was better in patients in internal wards. Furthermore, criticality of disease, lack of confidence in treatment, and inability to think rationally reduced the sense of control, thus creating a feeling of dependence on others and a threatened state of patient dignity (1).

The present study found a significant relationship between gender and dignity, with women feeling generally less dignified, and this relationship was pronounced in the social support dimension. Previous studies have also indicated the existence of a relationship between gender, social support, and depression (22). The women in this study suffered from depression and mental distress more than men. Women’s social role and negative stresses, like social conflicts, and the negative responses they receive from their social networks increases their vulnerability to the adverse effects of stress. These factors explain the difference in mental disorders between the sexes (23). 

The importance of social relationships as a feel-good factor in humans has been proven. Social support is a well-known concept in social sciences, health services and so on, and many studies have associated it with reduced mental disorders, emphasizing the role of gender as a social parameter (22). Hospitalization is associated with numerous physical and mental problems, which cause a feeling of need for more support. In Iran, most women are not employed outside of their homes and have limited access to social support networks, and thus feel they enjoy less support than men. Research indicates that reduced social support also affects physical health, and it may even increase mortality rate and functional impairments (24, 25).

One factor that may cause women’s perception of social support to differ from that of men is the type of support that they receive from each other when faced with stressful situations and health problems. A study by Neff and Karney also demonstrated that in stressful situations, women support their spouses more (26), and women have a greater tendency to preserve family relationships and provide support for family members (27, 28). Compared to men, women are more sensitive to lack of support, and naturally benefit more when they receive positive support (23).

Results of the present study demonstrated significant relationships between the level of patient satisfaction with household income and symptom distress, existential distress and dependency. Socio-economic status is one of the most essential social aspects of diseases (29). People in lower income brackets experience greater feelings of dependency and existential distress due to disease symptoms, and reduced feelings of social support and peace of mind. Brock asserted that poor physical health is both the cause and effect of disease, poverty, and poor lifestyle. Sick people become poor and even poorer with the loss of their jobs and income (30). The existential distress of patients with low income may result from the fact that poverty is associated with feelings of incapacity (31). Fear, insecurity, dependency, depression, anxiety, shame, despair, isolation, and powerlessness are non-quantitative emotions expressed by low-income patients (32). Low income equates to the inability to access food sources, obtain health services, and find employment; it further correlates with increased divorce and crime rates, and lack of skill and training (30), which can affect certain dimensions of human dignity. Some studies associate low family income with symptoms such as high blood pressure (29). The present study found that poor patients suffered increased symptom distress. It also found that low income strongly correlates to lower quality care and reduced respect, and people with lower incomes are forced into early discharge from the hospital regardless of the due course of treatment. Thus the vicious cycle of hospitalization, discharge, and prolonged illness continues (32, 33).

The present study found an inverse relationship between household size and peace of mind, so that peace of mind increased with reduced household size. Peace of mind and feelings of physical and mental well-being greatly affect both physical and mental health. Although results of other studies indicate that marriage can increase peace of mind and provide mutual support between spouses, having children and the consequently larger household sizes have no effect (or a negative effect) on peace of mind and the feeling of well-being (34). Kandel et al. found that couples without children or with children that are independent have better mental conditions than parents in larger households. Large household size reduces adult patients’ feelings of well-being and peace of mind (especially when ill or hospitalized) in two ways: first, a larger number of people in the family means more financial burdens and less economic welfare; second, it reduces parents’ emotional support of one another (35). Ross et al. found that in the presence of sufficient family income and proper supports and services, a higher number of children and consequently larger household size can positively affect parents’ mental status. Furthermore, this study asserted that at a similar income level, more populated households bear higher economic pressures, which increases depression in both men and women. Poor socio-economic status and a high number of children cause a low level of social support, which in turn increases depression and other psychological problems. All of these factors can reduce patients’ feelings of well-being and peace of mind and increase their psychological distress (36, 37).

The present study further observed an inverse correlation between age and the status of dignity. There is strong evidence that dignity is a serious concern for older people, as it is a multifaceted concept, encompassing identity (self-esteem, self-respect, and honesty), human rights (equality, choice), and autonomy. Consequently, when drastic changes are accompanied by increasing age, the aforementioned factors get involved (20). Social role, status and the smaller presence of older people in the society lead to reduced feelings of dignity in the elderly. Stratton and Tadd demonstrated that older people considered old age a period of deterioration in physical, mental, economic, and independence states (38). Loss of self-esteem may occur because of lack of support or decision-making rights, sentiments related to being treated as an object, or absence of social uniformity resulting from the inability to trust others (20). Therefore, national and international social and health care policies for the elderly must emphasize observation of their rights and maintaining their dignity, including increased public awareness and support services. In this regard, respectful treatment of patients by hospital staff, especially nurses, is extremely important (38, 20).

Current study results can serve to inform health policy makers about the appropriate planning and training of staff, especially nurses, to observe the following while providing health services; consideration for human dignity; respect for values, cultural and religious beliefs; honesty, justice and good manners; freedom from various forms of bias, including ethnic, cultural, religious, disease type, and gender; and current knowledge and practices. Moreover, the education of health service practitioners on preserving patient dignity must include in-depth learning opportunities.

One limitation of the present study was that the researcher had to complete the questionnaires for illiterate patients, which may have affected participants’ responses, especially in areas of economy and insurance, where people may have overstated their answers. Another limitation was that this study was conducted in hospitals affiliated with one university, and therefore the results may not be generalized to patients in other facilities. Thus, we recommend further studies in wider dimensions.

## Conclusion

Results of this study revealed an almost favorable status of human dignity in hospitals affiliated with one of the largest universities in Iran. Appropriate treatment setting and the proper behavior of hospital staff, especially nurses, are influential in increasing patients’ feelings of self-esteem and respect. Inappropriate patient dignity can affect patient outcome, the recovery process, and return to normal life, and therefore we recommend that maintaining human dignity and the factors influencing it be considered in the treatment of patients. 
